# The role of hematological parameters and systemic immune-inflammation index in the management of symptomatic gestational hydronephrosis

**DOI:** 10.1590/1806-9282.20260594

**Published:** 2026-10-05

**Authors:** Ubeyd Sungur, Kağan Toksal, Gül Şüheda Sungur, Deniz Noyan Özlü, Mithat Ekşi, Ali Emre Fakir, Alper Bitkin

**Affiliations:** 1 University of Health Sciences, Bakırköy Dr. Sadi Konuk Research and Training Hospital, Department of Urology – Istanbul, Turkey.; 2 University of Health Sciences, Bakırköy Dr. Sadi Konuk Research and Training Hospital, Department of Obstetrics and Gynecology – Istanbul, Turkey.

**Keywords:** Hydronephrosis, Pregnancy complications, Ureteral obstruction, Inflammation, Biomarkers

## Abstract

**OBJECTIVE:**

The aim of this study was to investigate the clinical, imaging, and hematological parameters predicting the necessity of surgical intervention in pregnant women presenting with symptomatic gestational hydronephrosis.

**METHODS:**

We retrospectively evaluated 192 pregnant women diagnosed with symptomatic hydronephrosis between January 2015 and September 2024. Patients were divided into Group 1 (conservative management, n=138) and Group 2 (surgical intervention via double-J stent or percutaneous nephrostomy, n=54). Demographic data, clinical findings, degree of hydronephrosis, urinalysis, and complete blood count parameters—including C-reactive protein, neutrophil-to-lymphocyte ratio, platelet-to-lymphocyte ratio, and systemic immune-inflammation index—were compared. Logistic regression analysis was performed to identify predictors for surgery.

**RESULTS:**

A history of urolithiasis, prolonged pain, fever, severe hydronephrosis, pyuria, and elevated creatinine were significantly higher in Group 2 (p<0.05). White blood cell, neutrophil-to-lymphocyte ratio, and C-reactive protein levels did not differ significantly between the groups. However, platelet counts (p=0.020), platelet-to-lymphocyte ratio (p=0.038), and systemic immune-inflammation index (p=0.015) were significantly elevated in the surgical cohort. Univariate logistic regression analysis revealed that an elevated platelet count (p=0.039) and a higher systemic immune-inflammation index (p=0.004) were significant predictors. In the multivariate analysis, the systemic immune-inflammation index remained an independent predictor (p=0.009), whereas platelet count did not.

**CONCLUSION:**

While clinical presentation and the severity of hydronephrosis primarily indicate the need for surgical intervention, the systemic immune-inflammation index serves as a valuable, cost-effective auxiliary predictor among the hematological parameters.

## INTRODUCTION

Gestational hydronephrosis generally presents as a transient condition that does not require invasive treatment. It is well established that this condition is caused by the mechanical compression of the growing fetus on the ureter—predominantly on the right side—coupled with the relaxant effect of progesterone^1^. While it is frequently detected via ultrasonography during routine check-ups in asymptomatic pregnant women, it occasionally presents with flank pain. Given the restricted imaging modalities during pregnancy, the possibility of ureteral stones must always be kept in mind^2^. Particular attention is paid to identify the management method, as interventions performed during pregnancy or delayed treatments can adversely affect both the mother and the fetus^3^. Currently, the criteria for determining the treatment modality in symptomatic gestational hydronephrosis lack clearly defined boundaries.

A known history of stone disease and recent prior imaging, if available, can be important in the decision-making process for surgical intervention in this patient cohort. In cases presenting with signs of pyelonephritis, fever, pyuria, prolonged intractable pain, or renal impairment, surgical alternatives such as double-J (DJ) stent placement or percutaneous nephrostomy are considered^4^. A risk-benefit analysis is conducted in collaboration with the patient, taking the remaining time until delivery into account. Although clinical findings primarily constitute the determining factors, the predictive value of inflammatory markers in this patient group has become a subject of ongoing research^5,6^. While mild leukocytosis is considered a normal physiological change during pregnancy, the presence of severe leukocytosis and neutrophilia, along with elevated parameters such as C-reactive protein (CRP), may indicate an impending septic state and thus aid in clinical decision-making^7,8^. Furthermore, parameters including the neutrophil-to-lymphocyte ratio (NLR), platelet-to-lymphocyte ratio (PLR), and systemic immune-inflammation index (SII) have emerged as hematological markers widely investigated for their role in signifying inflammation across various diseases^9,10^.

In this study, we aimed to investigate the hematological markers that predict the necessity for surgical intervention by comparing the clinical, imaging, and hematological parameters of pregnant women evaluated for symptomatic hydronephrosis who either underwent surgical intervention or were managed with conservative treatment.

## METHODS

Pregnant women diagnosed with hydronephrosis via ultrasonography during routine obstetric follow-ups or presentations to the emergency department, and who were subsequently referred to the urology department at our hospital between January 2015 and September 2024, were retrospectively reviewed. Based on patient history and consultation notes, symptomatic patients presenting with complaints such as pain, fever, or dysuria were included in the study. Patients with a solitary kidney, a history of renal transplantation, an immunodeficiency disorder, or missing essential laboratory data were excluded.

Upon admission, all patients underwent a complete blood count (CBC), biochemical evaluation including serum creatinine and CRP, urinalysis, and urinary ultrasonography. Patient histories were noted for gestational trimester, parity, presence of fever, duration of pain, and a history of urolithiasis. The patients were classified into two groups for comparison: Group 1 consisted of patients managed with conservative treatment, and Group 2 comprised those who underwent surgical intervention. Conservative management included fluid hydration, analgesic support, and antibiotherapy. Based on our institutional clinical practice, the indications for surgical intervention were defined as follows: (1) signs of pyelonephritis, defined as the coexistence of fever, pyuria, and hydronephrosis of any grade; (2) severe flank pain persisting for more than three days accompanied by hydronephrosis of any grade; or (3) severe hydronephrosis accompanied by elevated creatinine and/or CRP levels. Surgical interventions involved the placement of a 4.7-French (Fr) DJ stent under general anesthesia or sedation, or the insertion of an 8-Fr percutaneous nephrostomy tube under local anesthesia. The choice between the two procedures was determined by the surgeon according to the severity of hydronephrosis and patient preference. In the urinalysis, the presence of ≥10 leukocytes per high-power field (HPF) was defined as pyuria, and the presence of ≥4 erythrocytes per HPF was defined as hematuria.

The two groups were compared regarding demographic characteristics, clinical findings, the degree of hydronephrosis, and laboratory values. Subsequently, hematological parameters were subjected to logistic regression analysis to identify predictors for the necessity of surgical intervention.

### Statistical analysis

Categorical data were presented as frequencies and percentages. The Kolmogorov-Smirnov test was utilized to evaluate the normal distribution of numerical data. Normally distributed continuous variables were expressed as mean±standard deviation, whereas non-normally distributed continuous variables were reported as median and interquartile range (IQR). The Student’s t-test was used to compare normally distributed numerical data, and the Mann-Whitney U test was applied for non-normally distributed data. The frequencies of categorical variables were compared using Pearson’s chi-square test and Fisher’s exact test. Univariate logistic regression analysis was performed to determine the predictive value of hematological parameters for surgical intervention. Parameters found to be significant in the univariate analysis (platelet count and SII) were evaluated for multicollinearity by calculating the variance inflation factor (VIF) and were subsequently entered into a multivariate logistic regression model. Receiver operating characteristic (ROC) curve analysis was then performed to assess the discriminative ability of the SII, and the area under the curve (AUC) with its 95%CI, the optimal cut-off value (determined by the Youden index), sensitivity, and specificity were calculated. A p-value of <0.05 was considered statistically significant. All statistical analyses were performed using the Statistical Package for the Social Sciences version 26.0 (IBM SPSS Statistics; IBM Corp., Armonk, NY, USA).

## RESULTS

In our study evaluating 192 pregnant women, the patients were divided into two groups: those managed with conservative treatment (Group 1, n=138) and those who underwent surgical treatment (Group 2, n=54). Within Group 2, DJ stent placement was performed in 36 patients (66.7%), while 18 patients (33.3%) underwent percutaneous nephrostomy insertion. The mean age of the total cohort was 26.6±5.1 years. Age, trimester of pregnancy, and primiparity rates did not differ significantly between the two groups (p>0.05). A known history of urolithiasis, the duration of pain before admission, and the presence of fever were found to be significantly higher in Group 2 (p=0.011, p=0.001, and p<0.001, respectively). While there was no significant difference between the groups regarding the laterality of hydronephrosis, the rate of severe hydronephrosis was considerably higher in Group 2 (p<0.001).

Regarding the CBC parameters, no significant differences were detected between the two groups in terms of white blood cell (WBC), neutrophil, and lymphocyte counts. However, the platelet count was notably higher in Group 2 compared to Group 1 (p=0.02). The median values of the NLR and CRP were higher in Group 2 than in Group 1; however, these differences did not reach statistical significance (p=0.154 and p=0.248, respectively). Conversely, PLR and SII values were remarkably higher in Group 2 in contrast to Group 1 (p=0.038 and p=0.015, respectively). Elevated creatinine levels and the presence of pyuria in urinalysis were significantly higher in Group 2, whereas no significant difference was observed between the groups concerning hematuria. The detailed comparison between the two groups is presented in Table 1.

**Table 1:** Comparison of demographic, clinical, and laboratory parameters of the patients between the conservative management and surgical treatment groups.

Parameters (mean±SD; %)	Total (n=192)	Group 1 “Conservative” n=138 (71.9)	Group 2 “Surgical” n=54 (28.1)	p-value
Age (years)	26.6±5.1	26.5±5.4	27±4.6	0.585
Trimester (n; %)				
First trimester	19 (9.9)	10 (7.2)	9 (16.7)	0.136
Second trimester	96 (50)	70 (50.8)	26 (48.1)	
Third trimester	77 (40.1)	58 (42)	19 (35.2)	
Primiparous (n; %)	114 (59.4)	81 (58.7)	33 (61.1)	0.759
History of urolithiasis (n; %)	41 (21.4)	23 (16.7)	18 (33.3)	**0.011**
Duration of flank pain (days), median (IQR)	3 (4)	2 (3)	3 (5)	**0.001**
Fever (n; %)	29 (15.1)	12 (8.7)	17 (31.5)	**<0.001**
Hydronephrosis (n; %)				
Grade 1–2	138 (71.9)	116 (84.1)	22 (40.7)	**<0.001**
Grade 3–4	54 (28.1)	22 (15.9)	32 (59.3)	
Laterality of hydronephrosis (n; %)				
Right	118 (61.5)	87 (63)	31 (57.4)	0.651
Left	35 (18.2)	23 (16.7)	12 (22.2)	
Bilateral	39 (20.3)	28 (20.3)	11 (20.4)	
White blood cells (×10^3^/μL)	13.6±4.3	13.2±4.2	14.5±4.3	0.074
Neutrophil count (×10^3^/μL)	10.9±4.1	10.6±3.9	11.7±4.5	0.106
Lymphocyte count (×10^3^/μL)	1.69±0.66	1.71±0.65	1.66±0.69	0.647
Platelet count (×10^3^/μL), median (IQR)	244 (78)	237 (77.7)	260.5 (68.5)	**0.020**
NLR, median (IQR)	6.2 (5.76)	5.88 (5.22)	7.16 (6.86)	0.154
PLR, median (IQR)	152.8 (86.8)	147.8 (84)	169.8 (95.9)	**0.038**
SII, median (IQR)	1,593 (1,625)	1,465 (1,259)	1,817 (2,141)	**0.015**
CRP (mg/L), median (IQR)	12 (33)	11 (43.7)	16.5 (27.7)	0.248
Creatinine (mg/dL), median (IQR)	0.5 (0.21)	0.49 (0.18)	0.56 (0.33)	**0.002**
Pyuria (n; %)	91 (47.4)	56 (40.6)	35 (64.8)	**0.002**
Hematuria (n; %)	160 (83.3)	112 (81.2)	48 (88.9)	0.196

SD: standard deviation; NLR: neutrophile to lymphocyte ratio; PLR: platelet to lymphocyte ratio; IQR: interquartile range; SII: systemic immune-inflammation index; CRP: C-reactive protein. Bold values indicate statistical significance (p<0.05).

According to the univariate logistic regression analysis conducted to investigate the hematological predictors for the necessity of surgical intervention, elevated platelet count and SII values (calculated by dividing by 100) were found to be statistically significant predictors (p=0.039 and p=0.004, respectively). Prior to the multivariate analysis, these two significant parameters were assessed for multicollinearity; the correlation between them was weak (r=0.20), and the variance inflation factors were low (1.09 for platelet count and 1.35 for SII), indicating no significant multicollinearity. When both parameters were entered into the multivariate logistic regression model, the SII remained an independent predictor of the need for surgical intervention (OR 1.033, 95%CI 1.008–1.058, p=0.009), whereas the platelet count lost its statistical significance (OR 1.003, 95%CI 0.999–1.008, p=0.125). The results of the logistic regression analyses are outlined in Table 2. In the ROC curve analysis, the SII showed a limited discriminative ability for predicting the need for surgical intervention, with an area under the curve (AUC) of 0.613 (95%CI 0.517–0.705). The optimal cut-off value was 2,865, yielding a sensitivity of 40.7% and a specificity of 87.7%. Figure 1 illustrates the graphical comparison of SII between the two groups. Figure 2 illustrates the ROC curve of the SII for predicting the need for surgical intervention.

**Table 2:** Univariate and multivariate logistic regression analysis of hematological parameters for predicting the need for surgical intervention.

	Univariate Analysis			Multivariate Analysis		
	OR	95%CI	p	OR	95%CI	p
WBC	1.067	0.993–1.146	0.078			
Neutrophil	1.063	0.986–1.146	0.109			
Lymphocyte	0.894	0.554–1.441	0.645			
Platelet	1.004	1.000–1.008	**0.039**	1.003	0.999–1.008	0.125
NLR	1.029	0.981–1.078	0.244			
PLR	1.004	1.000–1.008	0.070			
SII (per 100 units)	1.036	1.012–1.062	**0.004**	1.033	1.008–1.058	**0.009**
CRP	1.002	0.998–1.007	0.321			

OR: odds ratio; CI: confidence interval; WBC: white blood cell; NLR: neutrophil-to-lymphocyte ratio; PLR: platelet-to-lymphocyte ratio; SII: systemic immune-inflammation index; CRP: C-reactive protein. Bold values indicate statistical significance (p<0.05).

**Figure 1:**
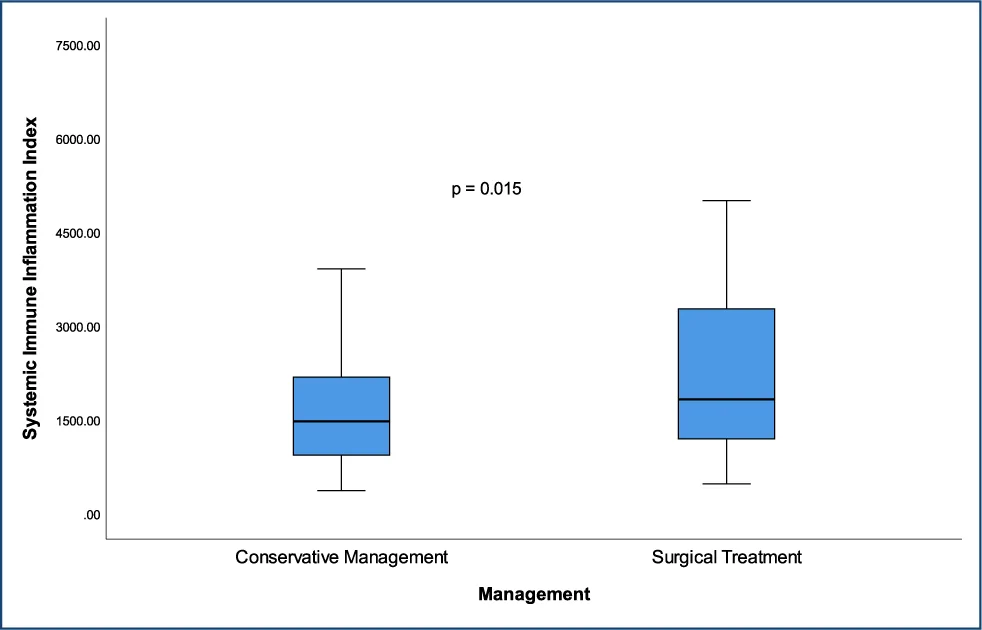
Comparison of the systemic immune-inflammation index between the conservative management group (Group 1) and the surgical treatment group (Group 2) (Mann-Whitney U test, p=0.015).

**Figure 2:**
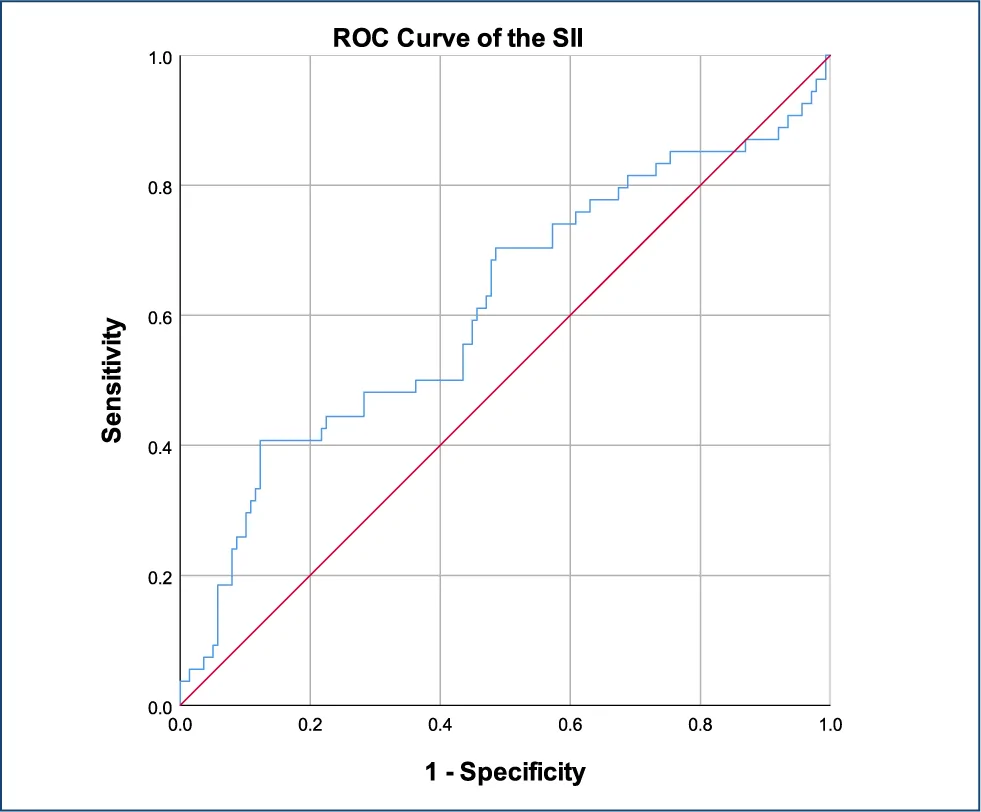
Receiver operating characteristic curve of the systemic immune-inflammation index for predicting the need for surgical intervention.

## DISCUSSION

Our study investigated the relationship between hematological parameters and the necessity for surgical intervention over 9 years by comparing pregnant women presenting with symptomatic hydronephrosis at our center who underwent either surgical procedures or conservative management. While clinical findings such as fever, prolonged pain, pyuria, and severe hydronephrosis on ultrasonography were observed at significantly higher rates in patients who underwent surgical intervention, the SII emerged as the most robust predictor among the evaluated blood parameters for the necessity of surgery.

During management of symptomatic hydronephrosis in pregnancy, the impact of inflammation on maternal health and the risk of infection must be carefully reviewed against the potential effects of interventional procedures on the fetus and overall obstetric outcomes^11^. Analyzing our results, we observed that clinical findings were the primary determining factors in patients who underwent percutaneous nephrostomy or DJ stent placement. Drainage is typically planned when findings of pyelonephritis, such as fever, pyuria, and flank pain, are accompanied by hydronephrosis indicative of urinary obstruction. Since computed tomography is contraindicated in this patient population, decision-making relies heavily on ultrasonography and clinical presentation. Consistent with this, our study found significantly higher rates of fever, prolonged pain, pyuria, and elevated creatinine levels in patients requiring surgical intervention. The elevated creatinine levels in the surgical group likely reflect the functional impact of urinary obstruction on renal drainage. Because glomerular filtration physiologically increases during pregnancy and lowers baseline serum creatinine, even values within or near the upper normal range may indicate subclinical renal impairment secondary to obstruction; a rising creatinine in this setting may therefore support the decision for decompression to preserve renal function. In a study examining pregnant women presenting with renal colic, He et al. reported that pain lasting longer than 4 days was a predictor for surgical intervention^12^. The severity of hydronephrosis also influences the clinician’s decision-making process by helping to differentiate pathological obstruction from physiological gestational hydronephrosis. The degree of hydronephrosis and anteroposterior pelvic diameter have been reported in the literature as parameters predicting the need for surgical intervention in pregnant women with hydronephrosis^13^. Our findings strongly support these existing data. Furthermore, it has been established that the presence of urolithiasis during pregnancy poses significant risks to both maternal and neonatal health^14^. Considering the recurrent nature of stone disease, we believe that in pregnant women with a history of urolithiasis who develop hydronephrosis, clinicians may be more inclined toward surgical intervention even if a stone is not directly visualized.

There are several studies in the literature evaluating the use of blood parameters in the decision-making process for symptomatic gestational hydronephrosis. Ercil et al. stated that elevated WBC counts and CRP levels, in conjunction with pain, are markers for the necessity of surgical intervention^5^. Conversely, a study published by Demir et al. found no significant difference in WBC and CRP elevations between patient groups managed surgically and those followed conservatively^15^. In our study, although WBC and CRP were higher in the surgical group, the difference did not reach statistical significance. It is well known that pregnancy-induced leukocytosis can occur, meaning WBC may not always accurately reflect an underlying pathological state^16^. Considering that it could be helpful without requiring additional laboratory testing, Bozkurt et al. investigated the NLR and identified that an elevated NLR could serve as an auxiliary parameter in the decision for surgical intervention^6^. Although the NLR was observed to be higher in the surgical group of our study, we did not detect a statistically significant difference. The SII, calculated by multiplying the NLR by the platelet count, has emerged as another valuable inflammatory marker. Previous literature has associated the SII with certain obstetric pathologies, including preeclampsia, early gestational diabetes, and threatened abortion^17,18,19^. In our cohort, the SII was found to be significantly elevated in patients undergoing surgery due to hydronephrosis and served as a significant predictor for the necessity of surgery. It is noteworthy that, while the individual leukocyte-based markers (WBC, CRP, and NLR) did not reach statistical significance, the composite SII did. This apparent paradox may be attributed to the fact that the SII additionally incorporates the platelet-driven component of the inflammatory response, which is not captured by the leukocyte-based indices alone, whereas the physiological leukocytosis of pregnancy may further attenuate the discriminative value of the WBC and NLR. Notably, although the PLR reached significance in the baseline group comparison (p=0.038), it did not retain significance in the univariate regression model (p=0.070), further underscoring the relative stability of the SII. Consistent with this, in the multivariate model the SII remained an independent predictor, whereas the platelet count alone did not, indicating that it is the combination of these components rather than the platelet count in isolation that carries the predictive signal. However, its limited discriminative ability on ROC analysis (AUC=0.613) indicates that the SII should be regarded as an adjunct to clinical and radiological assessment rather than a standalone predictor. It should also be emphasized that, although statistically significant, the odds ratios of these hematological parameters reflect modest per-unit increments (for instance, an OR of 1.033 per 100-unit increase in the SII). Accordingly, the value of the SII lies in its ability to add an accessible, objective, and quantifiable dimension to an otherwise clinically driven decision, complementing rather than competing with established clinical and radiological parameters.

In a normal, uncomplicated pregnancy, the platelet count may decrease slightly^20^. However, in the presence of inflammation, an increase in platelet count may be observed due to the activation of platelets and their role in the transport of cytokines^21^. In gestational hydronephrosis, the platelet count may be elevated due to inflammation secondary to obstruction, which consequently leads to higher calculated PLR and SII values. While the decision for surgical intervention in gestational hydronephrosis is primarily driven by clinical findings and imaging results, we believe that incorporating parameters such as SII into the evaluation process will provide valuable diagnostic assistance.

Our study has certain limitations. These include its retrospective design, single-center nature, and relatively small sample size. In addition, the multivariable model was restricted to hematological parameters and was not adjusted for clinical variables. However, it is particularly significant as it is the first study to investigate the relationship between the SII and symptomatic gestational hydronephrosis. We believe that multicenter prospective studies with larger patient cohorts are required to validate these findings further.

## CONCLUSION

The management of symptomatic gestational hydronephrosis relies predominantly on clinical presentation and ultrasound findings, with severe hydronephrosis, prolonged pain, fever, and pyuria strongly indicating the need for surgical intervention. However, our study demonstrates that the SII, an easily accessible and cost-effective marker, can serve as a valuable auxiliary predictor among the hematological parameters, while its limited discriminative ability indicates that it should complement rather than replace clinical and radiological assessment. As the first study to highlight the predictive value of the SII in this specific patient cohort, further studies are warranted to validate these findings.

## Data Availability

The datasets generated and/or analyzed during the current study are available from the corresponding author upon reasonable request.
